# Novel Insights Into Illness Progression and Risk Profiles for Mortality in Non-survivors of COVID-19

**DOI:** 10.3389/fmed.2020.00246

**Published:** 2020-05-22

**Authors:** Liang Shao, Xinyi Li, Yi Zhou, Yalan Yu, Yanan Liu, Minghui Liu, Ruixian Zhang, Haojian Zhang, Xinghuan Wang, Fuling Zhou

**Affiliations:** ^1^Department of Hematology, Zhongnan Hospital of Wuhan University, Wuhan, China; ^2^Department of Anesthesiology, Zhongnan Hospital of Wuhan University, Wuhan, China; ^3^Yunnan Center for Disease Control and Prevention, Kunming, China; ^4^Frontier Science Center for Immunology and Metabolism, Medical Research Institute, School of Medicine, Wuhan University, Wuhan, China; ^5^Evidence-Based and Translational Medicine, Department of Urology, Zhongnan Hospital of Wuhan University, Wuhan, China

**Keywords:** COVID-19, SARS-CoV-2, non-survivor, disease progression, complete clinical course

## Abstract

**Background**. The outbreak of COVID-19 has attracted the attention of the whole world. Our study aimed to describe illness progression and risk profiles for mortality in non-survivors.

**Methods**. We retrospectively analyzed 155 patients with COVID-19 in Wuhan and focused on 18 non-survivors among them. Briefly, we compared the dynamic profile of biochemical and immune parameters and drew an epidemiological and clinical picture of disease progression from disease onset to death in non-survivors. The survival status of the cohort was indicated by a Kaplan–Meier curve.

**Results**. Of the non-survivors, the median age was 73.5 years, and the proportion of males was 72.2%. Five and 13 patients were hospital-acquired and community-acquired infection of SARS-CoV-2, respectively. The interval between disease onset and diagnosis was 8.5 days (IQR, [4–11]). With the deterioration of disease, most patients experienced consecutive changes in biochemical parameters, including lymphopenia, leukocytosis, thrombocytopenia, hypoproteinemia, as well as elevated D-dimer and procalcitonin. Regarding the immune dysregulation, patients exhibited significantly decreased T lymphocytes in the peripheral blood, including CD3^+^T, CD3^+^CD4^+^Th, and CD3^+^CD8^+^Tc cells. By the end of the disease, most patients suffered from severe complications, including ARDS (17/18; 94.4%), acute cardiac injury (10/18; 55.6%), acute kidney injury (7/18; 38.9%), shock (6/18; 33.3%), gastrointestinal bleeding (1/18; 5.6%), as well as perforation of intestine (1/18; 5.6%). All patients died within 45 days after the initial hospital admission with a median survivor time of 13.5 days (IQR, 8–17).

**Conclusions**. Our data show that patients experienced consecutive changes in biochemical and immune parameters with the deterioration of the disease, indicating the necessity of early intervention.

## Introduction

In mid-December 2019, the outbreak of a novel coronavirus pneumonia in Wuhan, China, attracted the attention of the whole world (1–5). The virus was named as SARS-CoV-2 by the International Committee on Taxonomy of Viruses (ICTV), and the disease was called COVID-19 by the World Health Organization (WHO). By the 15th of April 2020, 837,513 confirmed cases, including 3,352 cases of death, of COVID-19 had been reported in China. Approximately 1397,354 confirmed cases and 134,734 deaths have been reported in countries outside China. The Chinese CDC has reported that the reproduction number (R_0_) of SARS-CoV-2 is 2.2, indicating that one COVID-19 patient can cause infection of 2~3 persons (6, 7). It suggests that SARS-CoV-2 has a strong transmission ability.

The most common clinical symptoms of COVID-19 are fever, dry cough, fatigue, and shortness of breath. Approximately 80% of COVID-19 patients are mild cases, and 20% are severe or critical cases. Although the estimated overall mortality is about 2%, over 50% of critically ill COVID-19 patients in Wuhan died due to multiple organ dysfunction and severe complications (8, 9).

A better understanding of the disease progression, especially for the severe or critically ill cases, is essential to the control and treatment of this epidemic. Herein, we have retrospectively studied 18 non-survivor cases in Wuhan and have presented the disease progression from their hospital admission to death. Our study might provide clues to a better understanding of the pathophysiology of COVID-19.

## Materials and Methods

### Patients

In this study, we retrospectively analyzed 155 patients with COVID-19 hospitalized in Zhongnan Hospital of Wuhan University (Wuhan, China) during the period between ~the 10th of January and the 8th of March, 2020. All participants met the criteria for clinical diagnosis based on The National Health Commission of China (NHCC) Guidelines (7th Edition) on COVID-19. Briefly, patients with two of the following clinical symptoms plus any epidemiological risk were suspected of COVID-19. Clinical symptoms included fever, cough, shortness of breath, imaging feature of pneumonia, as well as low or normal white blood cells or low lymphocyte count in peripheral blood. Epidemiological risks included a travel or residence history to Wuhan or neighboring regions in the past 2 weeks, close contact with confirmed patients with COVID-19, close contact with patients with respiratory symptoms, close contact with patients from regions with confirmed COVID-19 cases, or clustering cases. These patients then taken for laryngeal swabs test using a COVID-19 PCR Nucleic Acid Diagnostic Kit according to the manufacture's instructions.

Based on NHCC Guidelines (7th Edition), patients on the time of confirmed dignosis of COVID-19 are stratified: mild (i.e., mild clinical symptoms without imaging feature of pneumonia), ordinary (i.e., clinical symptoms, such as fever, cough, and with imaging feature of pneumonia), severe (i.e., dyspnea, respiratory frequency ≥30/min, blood oxygen saturation ≤ 93%, partial pressure of arterial oxygen to fraction of inspired oxygen ratio <300, and/or lung infiltrates > 50% within 24 to 48 h), and critically ill cases (i.e., respiratory failure, septic shock, and/or multiple organ dysfunction or failure).

This study was conducted according to the principles of Helsinki and approved by the Ethics Committee of Zhongnan Hospital of Wuhan University (No.2020063). We extracted the medical records in Zhongnan Hospital of Wuhan University. Three doctors participated in the collection and reviewing the clinical data. Due to the urgent need for this emerging epidemic, the requirements for informed consent from patients were waived.

### Statistical Analysis

Statistical analysis was performed with SPSS22.0. Data of normal distribution were indicated by mean ± standard deviation (SD), and statistical comparisons between hospital admission and death were performed using unpaired *t*-test. Correspondingly, data of abnormal distribution have been expressed as median and IQR, comparison between groups using Kruskal–Wallis test. The Kaplan–Meier curve was used to analyze the survival time of the patients. *P* < 0.05 was considered as statistical significance.

## Results

### General Characteristics of 155 Participants

A total of 155 patients with COVID-19 in hospitalization were retrospectively studied. Patients were stratified into four types based on NHCC Guidelines: mild (0%), ordinary (104/155; 67.1%), severe (25/155; 16.1%), and critically ill (26/155; 16.7%) (Supplementary Table 1). The median age of the cohort was 48 years (IQR, 33–63; range, 7–96 years), and 93 of them (93/155; 60.0%) were women. Eighteen patients were finally dead, while the remaining 137 patients were discharged from the hospital. Regarding to the hematological parameters for hospital admission, the white blood cell (WBC), neutrophil, and lymphocyte counts were significantly different among the ordinary, severe, and critically ill types (*p* = 0.000, *p* = 0.001, and *p* = 0.005, respectively). Notably, there was obvious increase in the WBC and neutrophil count as well as decreased lymphocyte count in critically ill patients. Moreover, severe and critically ill patients had elevated C-reactive protein (CRP), interleukin-6 (IL-6), highly sensitive troponin I (hsTnI), D-dimer, as well as β_2_-macroglobulin (β_2_-MG) levels compared to mild type patients. In terms of the immune dysregulation, with the progression of the disease, patients gained decreased CD3^+^T, CD3^+^CD4^+^Th, and CD3^+^CD8^+^Tc cell counts in the peripheral blood. Further correlation analysis showed that procalcitonin (PCT) was positively correlated with several parameters, including WBC, CRP, and IL-6 (Supplementary Table 2). Meanwhile, D-dimer was positively correlated with WBC, CRP, and hsTnI.

### Epidemiological and Clinical Characteristics of the Non-survivors

**Figure 1:**
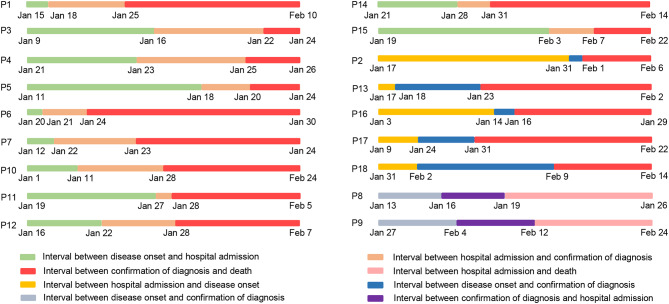
Time table for dates of illness onset, hospital admission, disease diagnosis, and death in 18 non-survivors with COVID-19. P is the abbreviation of Patient, and P1 represents Patient 1.

Next, we further investigated 18 non-survivor cases in this study. The epidemiological and clinical characteristics of these patients are summarized (Figure 1, Table 1). As shown in Table 1, the median age of the cohort was 73.5 years old (IQR, 65–78; range 29–96 years), and the proportion of males was 72.2%. Fifteen individuals (15/18; 83.33%) had underlying medical illnesses, including hypertension (10/18; 55.56%), cerebrovascular diseases (5/18; 27.78%), diabetes (4/18; 22.22%), chronic obstructive pulmonary diseases (3/18; 16.67%), renal diseases (3/18; 16.67%), malignancies (2/18; 11.11%), and chronic infectious diseases (2/18; 11.11%). In terms of the chronic infectious diseases, one individual was hepatitis B positive, and another was human immunodeficiency virus (HIV) positive. Of two patients with malignancies, one suffered from chronic lymphatic leukemia (CLL), and another had lung cancer. The most common clinical symptoms at disease onset were fever (15/18; 83.3%), cough (7/18; 38.9%), shortness of breath (5/18; 27.8%), myalgia or fatigue (3/18; 37.5%), and diarrhea (3/18; 37.5%). Abnormal chest computed tomographs (CT) or radiographs were observed among all patients. Typical chest CT of COVID-19 patients in hospitalization were bilateral ground glass opacity or multiple lobular areas of consolidation (Supplementary Figure 1). Seventeen patients showed bilateral involvement on chest radiographs in hospital admission (Table 2). In terms of the transmission routine, only one patient had a travel history of having been to the Huanan Seafood Market in Wuhan. As shown in Figure 1, five patients were likely to be hospital-acquired infections, and 13 were community-acquired infections. In addition, 16 patients were diagnosed as COVID-19 in hospitalization, while the other two patients were diagnosed in the outpatient service center. Regarding the disease stratification, 18 patients were critically ill cases. The interval between onset of symptoms and diagnosis of COVID-19 was 8.5 days (IQR, [4–11]). Meanwhile, the interval between hospital admission and death was 13.5 days (IQR, [8–17]) (Table 1, Figure 1).

**Table 1:** Epidemiological and clinical characteristics of non-survivors with COVID-19 in the study.

**Variables**	**All patients (*n* = 18)**
**Median ages (years)**	73.5 (29–96)
**Sex-*****n*** **(%)**	
Male	13 (72.2)
Female	5 (27.8)
**Disease stratification-*****n*** **(%)**	
Mild type	0
Ordinary type	0
Severe type	0
Critically ill type	18 (100)
**Underlying medical illness-*****n*** **(%)**	
Hypertension	10 (55.6)
Diabetes	4 (22.2)
Cerebrovascular disease	5 (27.8)
Renal disease	3 (16.7)
Carcinoma	2 (11.1)
Chronic obstructive pulmonary disease	3 (16.7)
Chronic infectious disease	2 (11.1)
Autoimmune disease	1 (5.6)
**Clinical symptoms-*****n*** **(%)**	
Fever	15 (83.3)
Cough	7 (38.9)
Expectoration	2 (11.1)
Sore throat	2 (11.1)
Myalgia or fatigue	3 (16.7)
Diarrhea	3 (16.7)
Headache	1 (5.6)
Shortness of breath	5 (27.8)
Haematemesis	1 (5.6)
Vomiting	1 (5.6)
**Co-infection status-*****n*** **(%)**	
Mycoplasma	1 (5.6)
Chlamydia	0
Influenza A	0
Influenza B	2 (11.1)
Respiratory syncytial virus	1 (5.6)
Adenovirus	1 (5.6)
Parainfluenza virus	1 (5.6)
Klebsiella pneumoniae	1 (5.6)
ESBL-producing Escherichia coli	1 (5.6)
Candida albicans	1 (5.6)
Acinetobacter baumannii	1 (5.6)
**Travel history to Huanan Seafood Market-** ***n*** **(%)**	1 (5.6)
**Interval between onset of symptoms and diagnosis of COVID-19 (days)**	8.5 (4–11)
**Interval between hospital admission and death (days)**	13.5 (8–17)

*Values are numbers (percentage) unless stated otherwise*.

**Table 2:** Laboratory characteristics of non-survivors with COVID-19 on the admission date to the death date in Zhongnan Hospital of Wuhan University.

**Variables**	**Normal range**	**Day of hospital admission**	**Day of death**	***P*-value**
**Complete blood count**				
White blood cell count (× 10^9^/L)	3.5–9.5	9.26 ± 7.71	15.45 ± 8.22	0.041
^#^Lymphocyte count (× 10^9^/L)	1.1–3.2	0.77 (0.38–1.29)	0.44 (0.34–0.84)	0.491
^#^Neutrophil count (× 10^9^/L)	1.8–6.3	4.66 (3.59–7.24)	12.41 (9.09–17.09)	0.008
Monocyte count(× 10^9^/L)	0.1–0.6	0.52 ± 0.28	0.50 ± 0.38	0.785
Monocyte (%)	3–10	6.82 ± 3.29	3.32 ± 2.25	0.001
Hemoglobin (g/L)	130–175	118.57 ± 32.49	109.47 ± 27.48	0.056
Platelet count (× 10^9^/L)	125–350	177.50 ± 110.57	115.28 ± 80.92	0.008
^#^Eosinophil count (× 10^9^/L)	0.02–0.2	0.005 (0–0.02)	0.015 (0–0.09)	0.766
^#^Basophil count (× 10^9^/L)	0–0.06	0.02 (0.01–0.03)	0.03 (0.02–0.08)	0.270
**Biochemical test**				
Total plasma protein (g/L)	65–85	63.23 ± 7.23	56.88 ± 8.30	0.011
^#^Globulin (g/L)	20–30	30.30 (28.10–31.90)	30.20 (25.00–33.00)	0.270
Albumin (g/L)	40–55	34.89 ± 7.90	26.98 ± 4.64	0.001
^#^Alanine aminotransferase (U/L)	9–50	39.00 (16.00–48.00)	41.50 (12.00–72.00)	0.491
^#^Aspartate aminotransferase (U/L)	15–40	50.00 (26.00–66.00)	62.00 (30.00–243.00)	0.491
^#^Blood urine nitrogen (mmol/L)	2.8–7.6	9.27 (6.68–14.41)	12.17 (9.42–23.70)	0.270
^#^Creatinine (μmol/L)	64–104	88.25 (76.70–123.70)	123.60 (70.80–328.70)	0.270
Uric acid (μmol/L)	208–428	392.08 ± 110.97	362.74 ± 186.04	0.570
^#^Lactate dehydrogenase (U/L)	125–243	474 (420–654)	560 (438–657)	0.992
^#^Brain natriuretic peptide (pg/mL)	<100	146.20 (59.85–328.45)	223.30 (66.60–860.40)	0.979
^#^Creatinine kinase-MB (U/L)	<171	32 (20–44)	80 (55–150)	0.627
^#^Highly sensitive troponin I (pg/mL)	0–26.2	21.5 (11.3–115.4)	69.2 (26.5–208.5)	0.290
^#^Potassium (mmol/L)	3.5–5.3	4.16 (3.87–4.55)	5.05 (4.30–5.84)	0.046
Sodium (mmol/L)	137–147	140.69 ± 11.01	138.02 ± 7.87	0.306
**Inflammatory profile**				
^#^Procalcitonin (ng/mL)	<0.05	0.64 (0.11–2.75)	4.58 (1.48–11.48)	0.032
^#^C-reactive protein (mg/L)	0–10	86.90 (27.36–160.55)	179.70 (136.10–322.40)	0.292
**Coagulation profile**				
^#^Prothrombin time (s)	9.4–12.5	12.20 (11.50–13.40)	14.75 (12.80–16.70)	0.022
^#^Activated partial thromboplastin time (s)	21.5–36.5	28.35 (26.10–31.40)	32.15 (29.50–38.60)	0.057
^#^Thrombin time (s)	10.3–16.6	14.85 (14.10–15.50)	16·45 (15.70–19.70)	0.002
Fibrinogen (mg/dL)	238–498	407.94 ± 110.16	427.11 ± 153.36	0.670
^#^D-dimer (mg/L)	0–500	492.50 (273.00–2139.00)	3542.50 (2797.00–10929.00)	0.002
**Blood gas analysis**				
SaO_2_	0.95–0.99	0.91 ± 0.09	0.69 ± 0.29	0.042
PCO_2_ (mmHg)	35–45	32.64 ± 11.34	62.63 ± 19.59	0.001
PO_2_ (mmHg)	83–108	73.1 ± 33.04	59.43 ± 24.34	0.368
PH	7.35–7.45	7.41 ± 0.09	7.05 ± 0.22	<0.001
BE (mmol/L)	−2.3–+3	−3.94 ± 6.24	−11.47 ± 10.70	0.086
${\text{HCO}}_{3}^{-}$ (mmol/L)	21.4–27.3	20.02 ± 6.05	17.57 ± 8.13	0.486
**Bilateral involvement on chest Radiographs—*****n*** **(%)**	NA	17 (94.44)	18 (100)	–

# *The data of abnormal distribution is expressed as median and IQR. NA, not applicable; SaO_2_, arterial oxygen saturation; PaCO_2_, partial pressure of carbon dioxide; PaO_2_, partial pressure of oxygen; BE, base excess*.

*Values are mean ± SD unless stated otherwise*.

### Co-infectious Status

In terms of co-infection status, five patients were co-infected with other pathogens, including one with mycoplasma (1/18; 5.6%), two with influenza B (2/18; 11.1%), one with respiratory syncytial virus (1/18; 5.6%), one with adenovirus (1/18; 5.6%), as well as one with parainfluenza virus (1/18; 5.6%) (Table 1). In addition, one showed extended-spectrum β-lactamase-producing Escherichia coli and Acinetobacter baumannii positive in sputum culture, one exhibited multidrug-resistant-Candida- albicans positive in urine culture and one had multidrug-resistant-Klebsiella-pneumoniae positive in sputum culture. Extremely high level of procalcitonin were observed on the death date compared with the admission date (median, 4.58 [IQR, 1.48–11.48] vs. 0.64 [IQR, 0.11–2.75, *p* < 0.05]).

### Laboratory Parameters

In order to present the disease progression of non-survivors, we collected and compared their laboratory data on the admission date with the death date. As shown in Table 2, patients exhibited a significant increase in WBC and neutrophil counts in the peripheral blood on the death date than the admission date (mean ± SD, 15.45 ± 8.22 vs. 9.26 ± 7.71, *p* < 0.05; median, 12.41 [IQR, 9.09–17.09] vs. 4.66 [3.59–7.24], *p* < 0.01), while the platelet count dramatically decreased (mean ± SD, 115.28 ± 80.92 vs. 177.50 ± 110.57, *p* < 0.01). It is worth mentioning that the majority of the cohort exhibited remarkably decreased lymphocyte count both on the admission date and the death date. Biochemical data showed that total plasma protein and albumin on the death date were decreased compared with that of the admission date (mean ± SD, 56.88 ± 8.30 vs. 63.23 ± 7.23, *p* < 0.05; 26.98 ± 4.64 vs. 34.89 ± 7.90, *p* = 0.001).

In terms of coagulation parameters, prothrombin time (PT) and thrombin time (TT) on the death date were significantly longer than the admission date (median, 14.75[IQR, 12.80–16.70] vs. 12.20[IQR, 11.50–13.40], *p* < 0.05; 16.45 [IQR, 15.70–19.70] vs. 14.85 [IQR, 14.10–15.50], *p* = 0.002). Moreover, an extremely high level of D-dimer was observed on the death date compared with the admission date (median, 3542.50 [IQR, 2797.00–10929.00] vs. 492.50 [IQR, 273.00–2139.00], *p* < 0.05). Blood gas analysis showed that the majority of the patients had a decreased PH value and increased level of PCO_2_ as well as decreased SaO_2_ on the death date (mean ± SD, 7.05 ± 0.22 vs. 7.41 ± 0.09, *p* < 0.001; 62.63 ± 19.59 vs. 62.63 ± 19.59, *p* = 0.001; 0.69 ± 0.29 vs. 0.91 ± 0.09, *p* < 0.05).

### Treatment and Disease Progression

The main intervention includes antivirus, antibacteria, antifungal, and glucocorticoid treatment as well as immune regulatory drugs and supportive treatment. As shown in Table 3, 94.4% of patients received antivirus therapy, such as oseltamivir, ribavirin, lopinavir and ritonavir, interferon α-2b, and abidol. During the hospitalization, all patients were given more than one of the antibacteria drugs, such as meropenem, tigecycline, biapenem, moxifloxacin, linezolid, as well as piperacillin tazobactam. Four patients (4/18; 22.2%) were administered with antifungal drugs based on the laboratory results and clinical symptoms. A total of 61.1% of patients received treatment with corticosteroids. Four (4/18; 22.2%) patients received gamma globulin, and two (2/18; 11.1%) were given thymosin. Regarding the supportive therapy, four (4/18; 22.2%) patients required blood transfusion, including red blood cells, platelets and plasma transfusion. A total of 18 patients required oxygen uptake, 15 (15/18; 83.3%) had non-invasive mechanical ventilation, and 11 (11/18; 61.1%) required invasive mechanical ventilation. Seven (7/18; 38.9%) patients were given continuous renal replacement therapy (CRRT), and one (1/18; 5.6%) required rescue therapy with extracorporeal membrane oxygenation (ECMO).

**Table 3:** Treatments and outcomes of 18 hospitalized COVID-19 patients in Zhongnan Hospital of Wuhan University, China.

	**All patients (*n* = 18)**
**Treatments—*****n*** **(%)**	
Antiviral therapy	17 (94.4)
Antibiotics	18 (100)
Antifungal	4 (22.2)
Corticosteroids	11 (61.1)
Gamma globulin	4 (22.2)
Thymosin	2 (11.1)
Blood transfusion	4 (22.2)
Oxygen uptake	18 (100)
Continuous renal replacement therapy	7 (38.9)
Non-invasive mechanical ventilation	15 (83.3)
Invasive mechanical ventilation	11 (61.1)
Extracorporeal membrane oxygenation (ECMO)	1 (5.6)
**Complications—*****n*** **(%)**	
ARDS	17 (94.4)
AKI	7 (38.9)
Shock	6 (33.3)
Acute cardiac injury	10 (55.6)
Gastrointestinal bleeding	1 (5.6)
Perforation of intestine	1 (5.6)

*Values are numbers (percentage) unless stated otherwise*.

By the end of the disease, most of the patients suffered from severe complications, including acute respiratory distress syndrome (ARDS) (17/18; 94.4%), acute cardiac injury (ACI) (10/18; 55.6%), acute kidney injury (AKI) (7/18; 38.9%), shock (6/18; 33.3%), gastrointestinal bleeding (1/18; 5.6%), as well as perforation of intestine (1/18; 5.6%).

Overall survival analysis showed that all patients died within 45 days after hospitalization, with the median survival time of 13.5 days (IQR, 8–17; range 2–44). Approximately 80% of patients died within the first 3 weeks (Supplementary Figure 2).

### The Dynamic Profile of Laboratory Data

**Figure 2:**
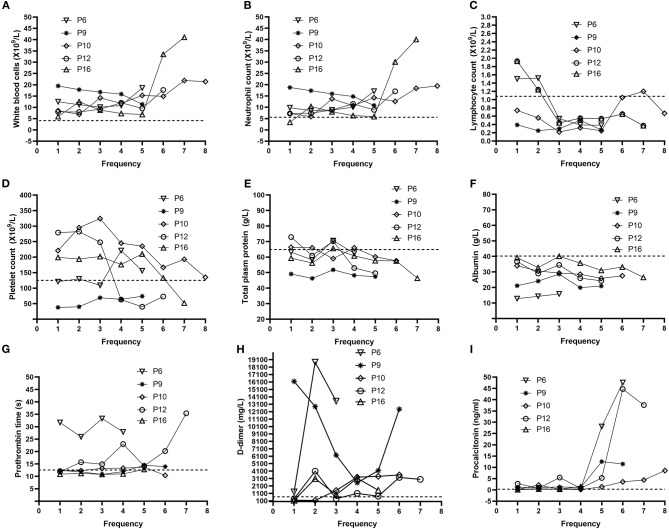
Dynamic profile of laboratory parameters for five representative non-survivors. Representative timeline charts from five non-survivors with COVID-19 were based on the frequencies of each test after hospitalization. The dash lines in black represent the normal upper limit of the parameters (white blood cell count, neutrophil count, prothrombin time, D-dimer, and procalcitonin) or lower limit of the other parameters (lymphocyte count, platelet count, total plasm protein, and albumin). P6, P9, P10, P12, and P16 represent Patient 6, Patient 9, Patient 10, Patient 12, and Patient 16, respectively. Dynamic changes in **(A)** white blood cells **(B)** neutrophil **(C)** lymphocyte **(D)** platelet **(E)** total plasm protein **(F)** albumin **(G)** Prothrombin time **(H)** D-dimer **(I)** Procalcitonin.

In order to determine the clinical features during COVID-19 progression, we tracked the dynamic changes in nine clinical laboratory parameters from the admission date to the death date. With the deterioration of the disease, the majority of patients gradually developed obvious lymphopenia as well as increased WBC and neutrophil counts (Table 2, Figure 2). Some patients had a gradually decreased platelet count. In addition, most patients exhibited several abnormal biochemical parameters during the disease progression, including decreased total plasma protein and albumin, prolonged prothrombin time, as well as extremely high level of D-dimer. Notably, with the development of the disease, the majority of patients showed significantly increased procalcitonin, suggesting the co-infection with bacteria.

### Supposed Time Schedule for the Disease Progression

**Figure 3:**
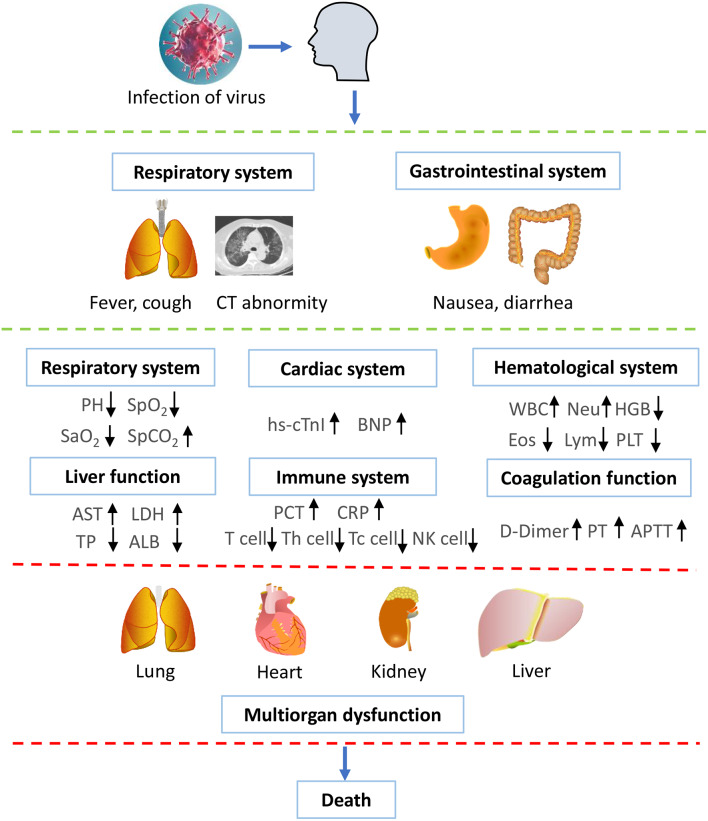
Supposed time schedule for the illness progression. People are occasionally infected with SARS-CoV-2 and present with clinical symptoms, such as fever, cough, diarrhea, and nausea. Subsequently, the laboratory results show that abnormality in chest CT and positive nucleic acid tests, confirming the diagnosis of COVID-19. The patient shows abnormal parameters in respiratory, cardiac, renal, liver, hematological, and immune systems. With the development of the disease, the patient might undergo electrolyte disturbance and DIC. By the end of the disease, the patient dies from multiple organs failure. WBC, white blood cell count; PLT, platelet count; HGB, hemoglobin; Eos, eosinophil count; Lym, lymphotye count; Neu, neutrophil count; AST, aspartate aminotransferase; LDH, lactate dehydrogenase; TP, Total plasma protein; ALB, albumin; hs-cTnI, highly sensitive troponin I; BNP, brain natriuretic pepetide; PCT, procalcitonin; CRP, C-reactive protein; PT, prothrombin time; APTT, activated partial thromboplastin time. ↑ represents increase; ↓ represents decrease.

We supposed the timeline for the disease progression of the people infected with SARS-CoV-2 (Figure 3). At the beginning, people are occasionally infected with SARS-CoV-2 and present with clinical symptoms, such as fever, cough, diarrhea, and nausea. After admission to the hospital, the patient shows abnormality in chest CTs, and SARS-CoV-2 nucleic acid tests are positive, confirming the diagnosis of COVID-19. Additionally, the patient shows abnormal parameters in respiratory, cardiac, renal, liver, hematological, and immune systems. With the progression of the disease, the patient might undergo electrolyte disturbance and disseminated intravascular coagulation (DIC). By the end of the disease, the patient dies from multiple organs failure.

## Discussion

In our study, we presented the clinical characteristics of 155 patients with COVID-19 and reported that patients had increased CRP, IL-6, hsTnI, D-dimer, and β_2_-MG with increased severity of the disease. Next, we focused on the 18 non-survivor cases and tried to draw a clear picture of complete course of the disease progression of them. We found that 13 patients were infected with SARS-CoV-2 in the community, while five patients were likely to acquire it during hospitalization. It should be noted that only one patient had a travel history of having been to the Huanan Seafood Market, which has been considered as one of the original places of the epidemic outbreak in Wuhan (10–13). Of the five patients with hospital-acquired infection of SARS-CoV-2, one (P2) received surgery because of perforation of the small intestine, which was followed by the development of clinical symptoms, such as fever and fatigue, after 9 days as well as the diagnosis of COVID-19 by nucleic acid test and imaging feature of pneumonia. Therefore, it is most likely that this patient acquired infection of SARS-CoV-2 in the hospital. In another special case, the patient (P13) had taken rehabilitation training in the hospital and developed fever and fatigue 2 days later. The following nucleic acid test and specific lung imaging supported his diagnosis of COVID-19. These aforementioned results suggest that the health authorities should be cautious about the risks of acquired infection of SARS-CoV-2 in hospitalized patients.

It has been reported that the overall death rate in COVID-19 is nearly 2% (8, 9). The major reasons for death are supposed to be the multiple organ dysfunction caused by the direct attack of SARS-CoV-2 (14–16). Herein, we suggest that the following crucial factors also might contribute to the death of the patients. Firstly, most of the non-survivors were older than 65 years and had underlying medical illnesses, resulting in poor tolerance to the virus attack. Second, our data showed that half of the patients were co-infected with respiratory viruses, bacteria, and mycoplasma. A total of 94.44% (17/18) of non-survivors exhibited high levels of procalcitonin on the day of death, indicating the high incidence of bacteria infection at the late stage of the disease. In a special case, the patient (P10) co-infected with ESBL-producing Escherichia coli and drug-resistant Acinetobacter baumannii in the intensive care unit, resulting in an unsatisfactory efficacy of the drugs. The doctors should also reconsider using antibiotics if there is a hint of bacteria co-infection, even though antibiotics should be used with caution to prevent drug resistance. Therefore, we suggest to the front line clinical practitioners that finding out the co-infected pathogens and taking the drug susceptibility test are beneficial to the treatment choice for COVID-19 patients. Third, no specific drugs to SARS-CoV-2 were confirmed by the official guideline at the very early stage of the epidemic outbreak. Therefore, the front line doctors treated the patients mainly by their own clinical experience. Fourth, some patients or relatives declined to receive treatments with invasive mechanical ventilation, CRRT, or ECMO due to old ages of the patients, leading to the loss of last chance for rescue. Actually, ECMO could be an alternative choice for some critically ill patients (17, 18).

Our study showed that there is obvious change in the T-cell subsets in the peripheral blood in the patients, with significantly decreased CD3^+^T, CD3^+^CD4^+^Th, as well as CD3^+^CD8^+^Tc NK cell counts, suggesting that the patients undergo significant immune dysregulation after infection with SARS-CoV-2. Our data is in accordance with Qin et al.'s report (19).

Our data have demonstrated that nine patients (9/18; 50.00%) did not have renal dysfunction, and 12 patients had normal levels of ALT and AST by the end of their death, suggesting that not all the patients underwent severe kidney and liver lesions by SARS-CoV-2. In contrast, the respiratory function was notoriously affected by this virus in all non-survivors with COVID-19. The majority of non-survivors (17/18; 94.44%) progressed to respiratory failure by the end. Our report is consistent with the study from Xu et al. where the most severely damaged organs caused by SARS-CoV-2 were lungs, and less severe lesions were in the heart and liver (14). It should be noted that one patient died with the acute coronary syndrome (ACS). SARS-CoV-2 can directly cause myocardial injury of the patients (14, 20). However, it is hard to differentiate whether the patient died from his original cardiac problem or the complications of SARS-CoV-2 infection. Furthermore, a majority of patients underwent a continuous decrease in the levels of total plasm protein and albumin, which might be due to the quick consumption of the body after SARS-CoV-2 infection. Therefore, timely and appropriate nutrition support for the patients is a necessary part of treatment. In terms of the coagulation function, our data are consistent with Tan et al.'s report, showing that COVID-19 patients with extremely high level of D-dimer and gradually prolonged prothrombin time and thrombin time (21). By the end, blood gas analysis of the patients showed increased PCO_2_, decreased PO_2_, as well as decreased PH values, suggesting severe obstruct ventilation disorder. These results are in accordance with the pathological report about a non-survivor with COVID-19, showing acute respiratory distress syndrome (ARDS) (14).

In conclusion, our data show that patients experienced consecutive changes in biochemical and immune parameters with the deterioration of the disease, indicating the necessity of early intervention for COVID-19 patients.

## Limitation of This Study

The main limitation of the present study is a relatively small number of non-survivor cases. Due to this limitation, the proportion of some clinical manifestations of the patients might be different from the reports from other cohort studies with large sample size. Second, we might show a relatively lower proportion of patients who co-infected with bacteria due to the limited sample size. Therefore, a cohort study with large numbers of patients is needed to verify our conclusions.

## Data Availability Statement

The raw data can be obtained with the permission of the corresponding author.

## Ethics Statement

The studies involving human participants were reviewed and approved by Zhongnan Hospital of Wuhan University. The ethics committee waived the requirement of written informed consent for participation. Written informed consent was not obtained from the individual(s) for the publication of any potentially identifiable images or data included in this article.

## Author Contributions

LS designed the project and wrote the manuscript. YZ, LS, YY, YL, ML, and XL collected the data. LS, RZ, and XL analyzed the data. XL and LS drew the pictures. HZ and FZ designed the project, provided professional guidance, and revised the manuscript. XW provided professional guidance.

## Conflict of Interest

The authors declare that the research was conducted in the absence of any commercial or financial relationships that could be construed as a potential conflict of interest.

## Supplementary Material

Supplementary Figure 1 Chest computed tomographic images of four non-survivors with COVID-19. **(A)** Representative CT image of a 78-year-old patient in hospitalization. Image shows ground-glass opacity in both lungs on day 4 after clinical symptoms onset. **(B)** Image of a 96-year-old patient shows ground-glass opacity and multiple lobular areas of consolidation in both lungs on day 30 after disease onset. **(C)** Representative CT image of a 78-year-old patient shows bilateral ground-glass opacity in lungs on day 14 after symptoms onset. **(D)** Representative CT image of a 66-year-old patient shows ground-glass opacity in both lungs on day 10 after symptoms onset.

Supplementary Figure 2 Survival curve of 18 non-survivor cases with COVID-19. A Kaplan–Meier curve was used to analyze the survival time of the patients.

Supplementary Table 1 Clinical characteristics of 155 COVID-19 patients in hospitalization.

Supplementary Table 2 Correlation analysis of clinical parameters in 155 COVID-19 patients.
