# Supplementary figures and images for: Novel Insights Into Illness Progression and Risk Profiles for Mortality in Non-survivors of COVID-19
Source: Front Med (Lausanne). 2020 May 22;7:246. doi: 10.3389/fmed.2020.00246 (PMC7256183; doi:10.3389/fmed.2020.00246)

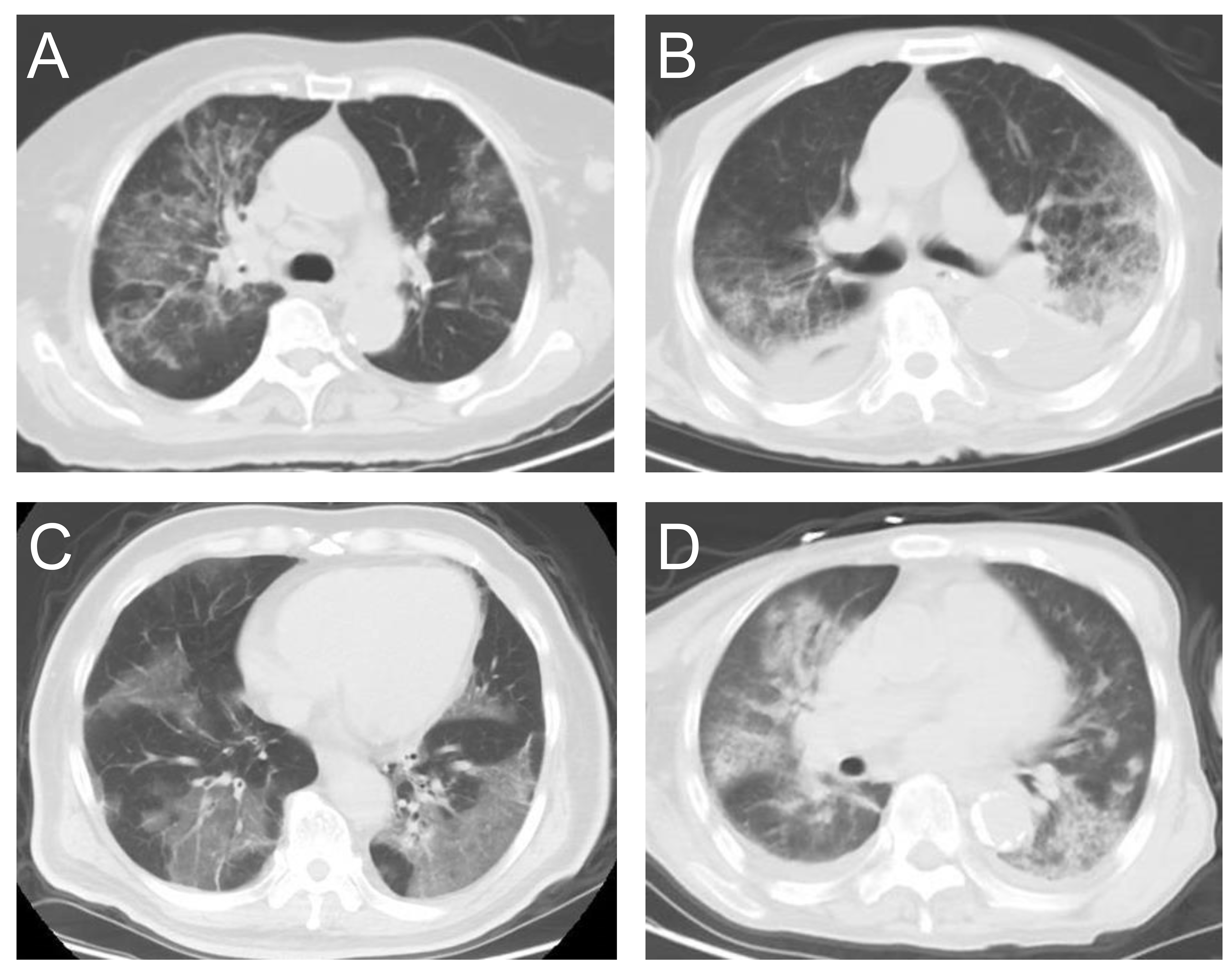

Supplement: Supplementary Figure 1 — Chest computed tomographic images of four non-survivors with COVID-19. (A) Representative CT image of a 78-year-old patient in hospitalization. Image shows ground-glass opacity in both lungs on day 4 after clinical symptoms onset. (B) Image of a 96-year-old patient shows ground-glass opacity and multiple lobular areas of consolidation in both lungs on day 30 after disease onset. (C) Representative CT image of a 78-year-old patient shows bilateral ground-glass opacity in lungs on day 14 after symptoms onset. (D) Representative CT image of a 66-year-old patient shows ground-glass opacity in both lungs on day 10 after symptoms onset. [file Image_1.TIF]

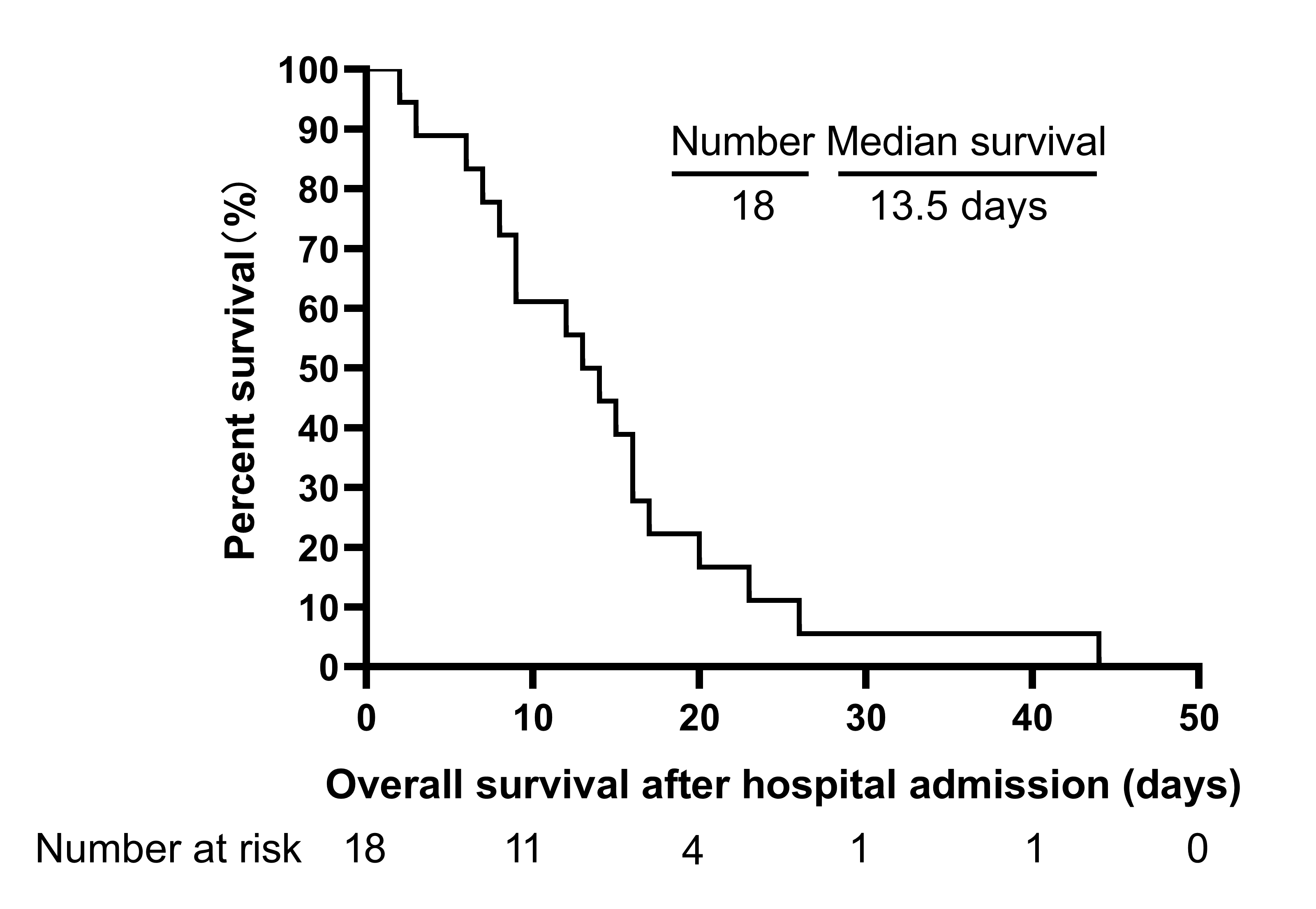

Supplement: Supplementary Figure 2 — Survival curve of 18 non-survivor cases with COVID-19. A Kaplan–Meier curve was used to analyze the survival time of the patients. [file Image_2.TIF]
