# Supplementary material for: Novel Insights Into Illness Progression and Risk Profiles for Mortality in Non-survivors of COVID-19
Source: Front Med (Lausanne). 2020 May 22;7:246. doi: 10.3389/fmed.2020.00246 (PMC7256183; doi:10.3389/fmed.2020.00246)
Supplement: Supplementary Table 1 — Clinical characteristics of 155 COVID-19 patients in hospitalization. [file Data_Sheet_1.docx]

**Supplementary Table 1. Clinical characteristics of 155 COVID-19 patients in hospitalization.**

| **Parameters** | **Total (N=155)** | **Ordinary (N=104)** | **Severe (N=25)** | **Critically ill (N=26)** | ***P* Value** |
| --- | --- | --- | --- | --- | --- |
| **Complete blood test** | | | | | |
| WBC, ×10^9^/L (NR: 3.5-9.5) | 6.16±4.41 | 5.65±3.05 | 4.74±2.89 | 9.86±7.96 | 0.000 |
| Neu, ×10^9^/L (NR: 1.8-6.3) | 4.65±4.23 | 4.09±2.80 | 3.73±2.71 | 7.96±7.96 | 0.001 |
| Lym, ×10^9^/L (NR: 1.1-3.2) | 0.99±0.61 | 1.10±0.66 | 0.81±0.35 | 0.71±0.43 | 0.005 |
| PLT, ×10^9^/L (NR: 125-350) | 186.80±71.49 | 194.50±66.64 | 173.50±72.64 | 85.77±16.82 | 0.151 |
| **Biochemical parameters** | | | | | |
| CRP, mg/L (NR: 0-10) | 49.26±63.03 | 32.02±41.01 | 61.47±63.75 | 107.81±95.02 | 0.000 |
| ^#^IL-6, pg/mL (NR: 0-7) | 20.51 (7.78-60.03) | 22.57(13.58-65.72) | 49.91 (23.57-69.66) | 95.47 (35.13-121.20) | <0.001 |
| ^#^hsTnI, pg/mL (NR: 0-26.2) | 7.50 (3.68-17.17) | 5.70 (2.20-9.40) | 8.20 (5.10-14.55) | 26.50 (16.90-153.00) | <0.001 |
| PT, s (NR: 9.4-12.5) | 12.66±1.67 | 12.49±1.61 | 13.00±1.50 | 13.04±1.97 | 0.173 |
| APTT, s (NR: 21.5-36.5) | 30.26±6.01 | 29.87±3.36 | 30.37±2.46 | 31.74±12.88 | 0.362 |
| Fib, mg/dL (NR: 238-498) | 435.05±88.71 | 438.45±78.70 | 428.80±90.98 | 427.58±121.66 | 0.797 |
| ^#^D-dimer, mg/L (NR: 0-500) | 273.00  (161.00-632.00) | 249.00  (146.50-600.00) | 239.00  (141.50-297.50) | 1304.00  (456.50-4707.50) | 0.002 |
| ^#^β_2_-MG, ug/L  (NR: 1000-3000) | 1755.60 (1515.90-2281.80) | 1755.60 (1510.65-2147.40) | 1697.90 (1507.80-2052.50) | 3836.40  (1743.90-5432.30) | 0.045 |
| **Lymphocyte subset analysis** | | | | | |
| CD3^+^T cell %  (NR: 38.56%-70.06%) | 66.55%±14.61% | 68.48%±12.95% | 67.91%±10.90% | 54.74%±21.18% | 0.014 |
| CD3^+^ T cell count  (NR: 805-4459) | 706.00±454.02 | 824.54±469.11 | 447.25±177.74 | 352.45±240.52 | 0.000 |
| CD3^+^CD4^+^Th cell %  (NR: 14.21%-36.99%) | 38.16%±11.74% | 38.65%±10.95% | 40.35%±12.92% | 33.12%±14.21% | 0.284 |
| CD3^+^CD4^+^Th cell count  (NR: 345-2350) | 428.13±340.34 | 504.39±367.90 | 255.00±88.37 | 208.00±145.11 | 0.004 |
| CD3^+^CD8^+^Tc cell count  (NR: 345-2350) | 280.73±203.64 | 327.29±212.36 | 179.33±119.96 | 141.64±110.01 | 0.003 |
| ^#^CD19^+^B cell count  (NR: 240-1317) | 127 (72-188) | 136 (85-95) | 82.5 (64-124) | 135 (34.50-188.50) | 0.194 |
| CD16^+^CD56^+^NK cell count  (NR: 210-1514) | 156.99±119.88 | 173.22±127.14 | 122.50±102.08 | 107.55±74.31 | 0.139 |

^#^ The data of abnormal distribution is expressed as median and IQR.

Abbreviations: NR, normal range; WBC, white blood cell; Neu, neutrophil; Lym, lymphocyte; PLT, platelet; CRP, C-reaction protein; PT, prothrombin time; APTT, activated partial thromboplastin time; Fib, fibrinogen; β2-MG, β2-microglobulin; NK cell, natural killer cell.

**Supplementary Table 2. Correlation analysis of clinical parameters in 155 COVID-19 patients**

|  | Correlation coefficient | WBC,  ×10^9^/L | Lym,  ×10^9^/L | CD3^+^T,  ×10^9^/L | PCT, ng/mL | CRP, mg/L | IL-6,  pg/mL | hsTnI, mg/L | D-dimer, mg/L |
| --- | --- | --- | --- | --- | --- | --- | --- | --- | --- |
| WBC,  ×10^9^/L | r | 1 | 0.110 | 0.360 | 0.426 | 0.519 | 0.001 | 0.389 | 0.435 |
|  | p |  | 0.251 | 0.016 | 0.010 | 0.000 | 0.992 | 0.004 | 0.000 |
| Lym,  ×10^9^/L | r | 0.110 | 1 | 0.234 | -0.148 | -0.192 | -0.208 | -0.184 | -0.068 |
|  | p | 0.251 |  | 0.035 | 0.305 | 0.027 | 0.028 | 0.103 | 0.408 |
| CD3^+^T,  ×10^9^/L | r | 0.360 | 0.234 | 1 | 0.076 | -0.447 | -0.184 | -0.035 | -0.106 |
|  | p | 0.016 | 0.035 |  | 0.744 | 0.000 | 0.103 | 0.838 | 0.346 |
| PCT, ng/mL | r | 0.426 | -0.148 | 0.076 | 1 | 0.167 | 0.472 | 0.024 | 0.050 |
|  | p | 0.010 | 0.305 | 0.744 |  | 0.053 | 0.002 | 0.879 | 0.726 |
| CRP, mg/L | r | 0.519 | 0.133 | -0.447 | 0.421 | 1 | 0.544 | -0.006 | 0.312 |
|  | p | 0.000 | 0.103 | 0.000 | 0.004 |  | 0.000 | 0.959 | 0.000 |
| IL-6,  pg/mL | r | 0.001 | -0.208 | -0.184 | 0.472 | 0.544 | 1 | 0.273 | -0.007 |
|  | p | 0.992 | 0.028 | 0.103 | 0.002 | 0.000 |  | 0.033 | 0.944 |
| hsTnI, mg/L | r | 0.389 | -0.132 | -0.035 | 0.024 | 0.456 | 0.273 | 1 | 0.397 |
|  | p | 0.004 | 0.231 | 0.838 | 0.879 | 0.005 | 0.033 |  | 0.000 |
| D-dimer, mg/L | r | 0.435 | -0.068 | -0.106 | 0.050 | 0.312 | -0.007 | 0.397 | 1 |
|  | p | 0.000 | 0.408 | 0.346 | 0.726 | 0.000 | 0.944 | 0.000 |  |

Abbreviations: WBC, white blood cell; Neu, neutrophil; Lym, lymphocyte; PLT, platelet; CRP, C-reaction protein; hsTnI, highly sensitive troponin I.
